# Competitiveness of Spanish Local Breeds

**DOI:** 10.3390/ani12162060

**Published:** 2022-08-12

**Authors:** José Perea, Ramón Arias

**Affiliations:** 1 Departamento de Producción Animal, Universidad de Córdoba, 14071 Córdoba, Spain; 2 Centro Regional de Selección y Reproducción Animal (CERSYRA), Instituto Regional de Investigación y Desarrollo Agroalimentario y Forestal de la Junta de Comunidades de Castilla-La Mancha (IRIAF), CERSYRA Valdepeñas, 13300 Ciudad Real, Spain

Spain is among the countries with the greatest diversity of local breeds, as a result of an extensive and heterogeneous cultural and agroecological heritage. The advancement of industrial farming, more focused on cost efficiency and food production for globalized markets, has progressively reduced the utility of local breeds. The loss of competitiveness of traditional farming systems linked to local breeds, and factors such as depopulation of rural areas, have expanded the list of local breeds at risk of extinction.

However, Spanish local breeds provide multiple transferable models to other environments on how to generate and make use of the competitive advantages based on consumer preferences, interests of society or attributes of the farming system, among others. In this sense, there are many related factors, including food safety, the circular economy, mitigation of environmental impacts and adaptation to climate change, animal welfare, higher quality, and sensory acceptance by consumers, with differentiation through market-oriented labelling statements. In fact, Spain is among the countries with the highest volume of production of differentiated quality schemes such as Organic Farming, Protected Geographical Indication (PGI) or Protected Designation of Origin (PDO).

The Spanish National Program for the Conservation, Improvement and Promotion of Livestock Breeds, contributes to the preservation and enhancement of native breeds, and is considered as one of its strategic priorities the sustainable use and the improvement of the profitability of breeds and their products, for which, among other actions, it has developed the “100% Autochthonous Breed” quality mark [1].

This Special Issue of *Animals*: “Competitiveness of Spanish Local Breeds”, collects a total of 12 manuscripts focused on a diversity of topics that are considered key to address the main challenges facing the livestock sector, such as adapting production systems to climate change, maintaining biodiversity and providing the necessary food without endangering the health of the planet.

Five papers deal with small ruminants, three of them focusing on goats, one on sheep, and one on both species. “*Selection Criteria for Improving Fertility in Spanish Goat Breeds: Estimation of Genetic Parameters and Designing Selection Indices for Optimal Genetic Responses*” [2] proposes a new selection index based on the age at first kidding and the interval between the first and second kidding as early selection criteria for female fertility in the breeds Florida and Payoya, which are two of the most representative Spanish dairy goat systems. The paper compares the genetic response of six selection indices based on several female fertility criteria, which are traits of interest for the economy and competitiveness of dairy goat farms that have not been previously considered in selection schemes. “*How Management System Affects the Concentration of Retinol and* *α-Tocopherol in Plasma and Milk of Payoya Lactating Goats: Possible Use as Traceability Biomarkers*” [3] examines the bioavailability of vitamins A and E in Payoya goats fed with different systems, and suggests the usefulness of both compounds as traceability biomarkers of the feeding system. Both compounds can also be useful as traceability biomarkers to discriminate Payoya kids from natural lactation if they are considered together with fat color and lipid oxidation, as proposed by “*Retinol and* *α-Tocopherol Contents, Fat Color, and Lipid Oxidation as Traceability Tools of the Feeding System in Suckling Payoya Kids*” [4]. “*GHG Emissions from Dairy Small Ruminants in Castilla-La Mancha (Spain), Using the ManleCO_2_ Simulation Model”* [5] presents an empirical model to estimate the carbon footprint of dairy small ruminants, considering the peculiarities of dairy farming of Spanish autochthonous breeds. The results show the contribution to climate change of different livestock practices, providing valuable information to plan and develop different strategies. “*Intramuscular Fatty Acids in Meat Could Predict Enteric Methane Production by Fattening Lambs*” [6] presents a precise and reproducible regression model that allows the estimation of methane production in Manchega lambs from the average body weight and the fatty acid profile of meat.

Two papers are focused on cattle, one on dairy farming and other on the Lidia breed. “*Analyses of Genetic Diversity in the Endangered ‘Berrenda’ Spanish Cattle Breeds Using Pedigree Data*” [7] evaluates the population structure of Berrenda en Negro and Berrenda en Colorado, two endangered Spanish breeds of great phenotypic and genotypic singularity. The results suggest the exchange of sires between herds and the monitoring of genetic contributions before carrying out any selective action to promote the conservation of genetic variability in both breeds. “*Beef from Calves Finished with a Diet Based on Concentrate Rich in Agro-Industrial By-Products: Acceptability and Quality Label Preferences in Spanish Meat Consumers*” [8] shows that fattening calves with a diet rich in non-edible fibrous local agro-industrial by-products for humans from southern Spain improves the color, flavor and tenderness of meat, increasing its acceptance by regular meat consumers. This paper also analyzes the importance of quality labels in building the preferences of Spanish consumers, finding that certification of origin, price and animal welfare are the most important attributes.

Published data on the Lidia breed is quite limited. “*Blood Biochemical Variables Found in Lidia Cattle after Intense Exercise*” [9] evaluates the biochemical and hormonal changes that occur in venous blood after the bullfight caused by stress and intense exercise. “*Value-Creating Strategies in Dairy Farm Entrepreneurship: A Case Study in Northern Spain*” [10] explores the different value creation strategies followed by dairy farmers. The manuscript identifies four different types of farms according to the main way of creating value: ecological, unique product, innovative and traditional.

The special issue has also articles dedicated to dog, horse and pig breeds. “*Study of Behavioural Traits in Can de Palleiro (Galician Shepherd Dog)*” [11] is the first scientific study focused on the behavior of Can de Palleiro, a former endangered Galician dog breed. “*Analysis of the Sustainability of Fattening Systems for Iberian Traditional Pig Production through a Technical and Environmental Approach*” [12] presents an economic evaluation of the environmental impact of “cebo de campo” versus “montanera” fattening as the main finishing strategies for the Iberian pig. Results show that it is possible to improve the sustainability of traditional Iberian pig farming through management strategies in both types of fattening. “*Assessment of Age Effects on Ovarian Hemodynamics Using Doppler Ultrasound and Progesterone Concentrations in Cycling Spanish Purebred Mares*” [13] evaluates the usefulness of the non-invasive Power Doppler imaging technique to monitor ovarian hemodynamics and blood flow adequacy of the corpus luteum in order to estimate the concentration of progesterone in the blood of Purebred Spanish mares.

The research performed on autochthonous Spanish breeds has not always reached all the impact and relevance that would correspond to the merit and scientific quality of the studies carried out. For this reason, this special issue has been an excellent opportunity to increase the international scientific impact of the Spanish autochthonous breeds and of the research groups that have been carrying out first-rate work with them.

## Data Availability

Not applicable.
